# A Case Report: First Long-Term Treatment With Burosumab in a Patient With Cutaneous-Skeletal Hypophosphatemia Syndrome

**DOI:** 10.3389/fendo.2022.866831

**Published:** 2022-05-06

**Authors:** Lea Maria Merz, Florian Buerger, Niels Ziegelasch, Martin Zenker, Ilse Wieland, Tobias Lipek, Tillmann Wallborn, Nicolas Terliesner, Freerk Prenzel, Manuela Siekmeyer, Katalin Dittrich

**Affiliations:** ^1^Department of Pediatric Nephrology and Pulmonology, University Hospital Leipzig, Leipzig, Germany; ^2^Boston Children’s Hospital, Harvard Medical School, Boston, MA, United States; ^3^Faculty of Medicine, University Hospital Magdeburg, Magdeburg, Germany; ^4^Department of Pediatric Nephrology, St. Georg Hospital, Leipzig, Germany; ^5^Department of Pediatric Nephrology, Charité Universitätsmedizin Berlin, Berlin, Germany

**Keywords:** burosumab, epidermal nevus syndrome, cutaneous-skeletal-hypophosphatemia-syndrome, hypophosphatemic rickets, hypophosphatemia

## Abstract

Epidermal nevus syndromes encompass a highly heterogeneous group of systemic disorders, characterized by epidermal nevi, and a spectrum of neuromuscular, ocular, and bone abnormalities. Cutaneous-skeletal hypophosphatemia syndrome (CSHS) constitutes a specific sub-entity in which elevated levels of fibroblast growth factor-23 cause hypophosphatemic rickets that are, to date, not amenable to causal therapy. Here, we report the first long-term follow-up of causal treatment with burosumab in a 3-year-old female patient with CSHS. 4 weeks after initiation of burosumab treatment, serum phosphate normalized to age-appropriate levels. Furthermore, long-term follow-up of 42 months revealed significant improvement of linear growth and gross physical functions, including respiratory insufficiency. Radiographic rickets severity as well as subjective bone pain were strongly reduced, and no side effects were observed over the course of treatment. In summary, we, here, report about a successful treatment of hypophosphatemic rickets in CSHS with burosumab over the time course of 42 months. In our patient, burosumab showed convincing efficacy and safety profile, without any loss of effect or increase of dose.

## Introduction

Epidermal nevus syndromes encompass a group of systematized mosaic disorders with wide phenotypic variability, characterized by epidermal nevi, and a spectrum of neuromuscular, ocular, and bone abnormalities (1). The group of epidermal nevus syndromes is characterized by poorly defined phenotypes, overlap of syndromes, lack of a unified classification, and a limited understanding of clinical progression. Therefore, the term ‘epidermal nevus syndrome’ is used inconclusively in the medical literature. Regarding their molecular basis, they are part of the wide spectrum of mosaic RASopathies (2). The RAS-mitogen-activated protein kinase (MAPK) pathway plays an important role in cell growth, differentiation, and survival through activation and regulation of various signaling proteins. RAS mutations with activating effects of different degree may occur either in the germline or as somatic events. Specific variants that have long been known as somatic mutations in cancer and are considered lethal in the germline may nevertheless survive in mosaicism. A specific sub-type of epidermal nevus syndromes is cutaneous-skeletal hypophosphatemia syndrome (CSHS). CSHS is characterized by elevated levels of fibroblast growth factor 23 (FGF23), leading to hypophosphatemic rickets accompanied by bone pain, fractures, scoliosis, and limb deformities. To date, the underlying molecular mechanism of FGF23 dysregulation in CSHS patients with *RAS* variants remains elusive. CSHS is not amenable to causal therapy, and, so far, patients receive symptomatic treatment with phosphate and calcitriol to achieve mineral homeostasis.

Burosumab, a recombinant human monoclonal antibody that binds FGF23, is a promising new drug to causally treat conditions with elevated levels of FGF23. However, burosumab is approved for patients with X-linked hypophosphatemia (XLH) and tumor-induced osteomalacia (TIO) since February and April 2018 in the European Union and the United States, respectively (3). To date, there is no systematic study on treatment of mineral homeostasis in pediatric CSHS patients. Very recently, a first pediatric patient with CSHS, and short-term treatment of burosumab over 12 months was reported (4). Furthermore, an ongoing phase 2 trial investigating the safety and efficacy of burosumab treatment in adult patients with TIO, also includes a single adult CSHS patient (5). Herein, we want to describe another case with this very rare condition and report about the first long-term off-label burosumab treatment of a pediatric CSHS patient. Observations of successful treatment and a significantly improved clinical condition like in our patient may promote the initiation of clinical trials with burosumab treatment in pediatric CSHS patients.

## Case Description

**Figure 1:**
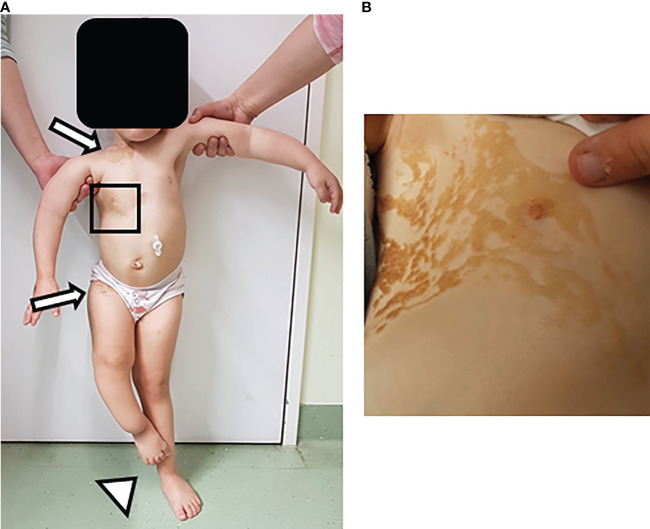
Skin lesions in patient with Cutaneous-skeletal hypophosphatemia syndrome. Physical examination of a three-year-old female patient showed multiple clinical findings, most prominently a linear nevus sebaceous. Additional features included asymmetric and age-inappropriate growth, accompanied by a leg length discrepancy, and a thoracic deformity with scoliosis. **(A)** Image shows overview image of the patient. Arrows point at the upper and lower end of the nevus sebaceous. Arrowhead points at the leg length discrepancy. **(B)** Image depicts inset of the nevus sebaceous of the upper right torso.

The patient is a three-year-old female who was referred to our outpatient nephrology clinic with a right-sided multicystic dysplastic kidney. Physical examination of the patient showed multiple clinical abnormalities, most prominently a linear, partially ulcerated nevus sebaceous covering the entire right upper body (**Figures 1A, B**). Furthermore, we noticed a hemangioma (0.6 x 0.4 x 0.3 cm) close to the right mandible. Additional features included asymmetric and delayed growth, accompanied by a leg length discrepancy, and a thoracic deformity with scoliosis. The medical history revealed daily, massive, generalized bone pain, affecting the whole body and strongly limiting the patient’s mobility and confining her to a wheelchair. Based on the syndromic phenotype, we initiated an extensive diagnostic work-up.

## A Figure or Table Showcasing a Timeline With Relevant Data From the Episode of Care


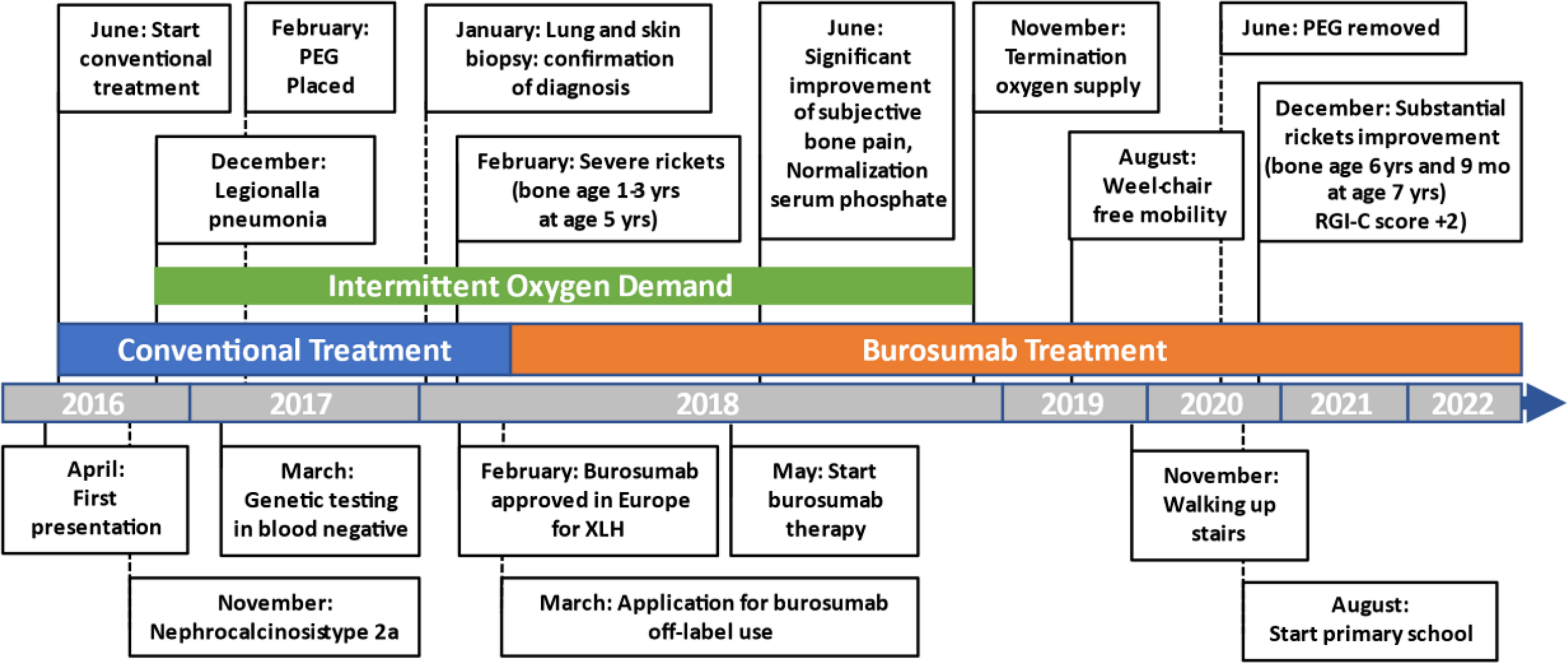


## Diagnostic Assessment, Details on the Therapeutic Intervention, Follow-Up and Outcomes, as Specified in the CARE Guidelines

**Figure 2:**
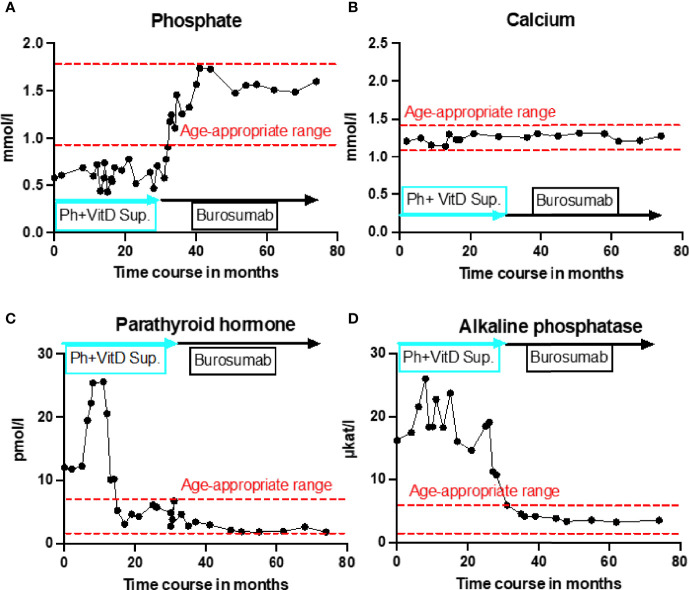
Timeline representation of laboratory values over 42 months of burosumab treatment. All graphs show individual laboratory values over the entire treatment course, starting from initial presentation at the age of 3.5 years until recently. Red dashed lines indicate age-appropriate norm range. Blue rectangle indicates conventional treatment with calcitriol and phosphate supplementation. Black rectangle indicates treatment with burosumab. **(A)** Serum phosphate levels in mmol/L showed no change under conventional treatment but rapidly normalized under burosumab treatment and remained in age-appropriate range throughout the entire follow up. **(B)** Calcium parameters in mmol/l constantly remained in the age-appropriate rage regardless of the treatment type. **(C)** Parathyroid hormone in pmol/l was severely increased under the first 12 months of conventional treatment and without treatment change decreased to the upper limit of age-appropriatre norm range. Under burosumab treatment further slight decline to lower end of age-appropriate range. **(D)** Alkaline phosphatase in μkat/l remained strongly increased under conventional treatment and showed a rapid normalization to the age-appropriate range under burosumab therapy.

**Figure 3:**
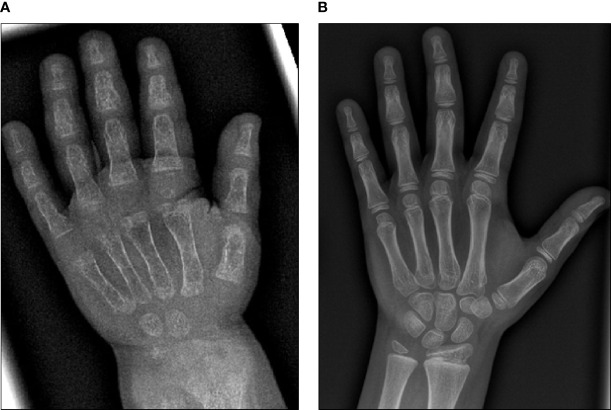
X-ray images of the left hand before and after 42 months of burosumab treatment. **(A)** X-ray image of the left hand at 3.5 years of age. Note gracile, undermineralized bone structure with loss of definition of the provisional calcification zone at the epiphyseal/metaphyseal interface and disorganization of the growth plate. Bone age was determined to be 1 year. **(B)** Follow-up X-ray image of the left hand at 8 years of age. According to the RGI-C score, a substantial improvement in ricket severity (RGI-C score +2) is noted. Overall skeletal maturity with a nearly age-appropriate bone age of 7.5 years was observed.

Initial blood analysis revealed profoundly reduced phosphate levels around 0.58 mmol/l (normal range 1.0-1.95 mmol/l) (**Figure 2A**), a reduced calcifediol level of 10 ng/ml (> 30 ng/ml) and highly increased FGF23 levels of >70.000 RU/ml (normal range 25-110 RU/ml). Osteocalcin and calcium were in the age-appropriate normal range (**Figure 2B**). Parathyroid hormone, as an important calcium and vitamin D regulator, was increased 12 pmol/l (normal range 1.6-6.9 pmol/l) (**Figure 2C**). As a marker for osteomalacia, bone specific alkaline phosphatase was tested and found to be significantly increased to 754 μg/l (normal range 37-97 μg/l) (**Figure 2D**). Renal function parameters: creatinine, urea and glomerular filtration rate, were unrestricted, and urine analysis showed increased phosphate excretion, indicating a decrease in tubular phosphate reabsorption. To assess the walking ability, we performed a six-minutes-walk test, revealing dramatically impaired skills with a walking distance of 15 meters. Radiographic examination revealed a gracile, undermineralized bone structure with loss of definition of the provisional calcification zone at the epiphyseal-/metaphyseal interface and disorganization of the growth plate (**Figure 3A**). Height parameters were persistently below the third percentile (height 77.5 cm, SDS -4.9), while bone age was determined to correspond to 1 year (at the age of 3 years). Abdominal ultrasound confirmed a right-sided multicystic dysplastic kidney, without other intra-abdominal malformations. After a legionella pneumonia our patient developed a persistent chronic oxygen demand. A CT scan of the lungs confirmed the severe thoracic deformity and additionally revealed a diffuse consolidation of the lung parenchyma, accompanied by ground glass opacifications in both lower lobes. A bronchoalveolar lavage did not show any signs of inflammation, pathogens, or alveolar hemorrhage. The combination of clinical and diagnostic findings, except lung involvement, was suggestive for an underlying epidermal nevus syndrome. A biopsy of the epidermal nevus was performed for histological and genetic examination. To investigate the oxygen demand and to exclude a parenchymal lung disease, as well as to determine the histology of the hemangioma, we also performed biopsies of the lung, and skin lesion at the right mandible within the same procedure. Histology of the skin biopsy of the epidermal nevus confirmed the clinical diagnosis of a nevus sebaceous. Ultra-deep next generation sequencing, yielding an average coverage of 5000x of the target regions, was performed on a DNA sample extracted from the biopsy material using a TruSeq™ Custom Amplicon Kit (Illumina) and sequenced on a MiSeq system (Illumina). This revealed a known pathogenic variant in *NRAS* (neuroblastoma RAS viral oncogene homolog), c.182A>G; p.Gln61Arg (NM_002524.5; 1079/5070 reads, 21.3%), thereby confirming a mosaic RASopathy. Histology of the lung biopsy showed an alveolar hypoplasia and neuroendocrine cell hyperplasia without further signs of diffuse parenchymal lung disease. Genetic testing of the lung tissue biopsy did not show the mosaic *NRAS* variant, that was detected in the nevus biopsy. To exclude neurological abnormalities, we performed a cranial MRI, revealing a single small arachnoid cyst as an incidental finding without further necessity for intervention. We immediately initiated a conventional therapy with oral phosphate und calcitriol supplementation to establish mineral homeostasis. The therapy with phosphate initially caused diarrhea, while the calcitriol supplementation was well tolerated. As a side effect of the calcitriol supplementation our patient developed nephrocalcinosis type 2a, that was detected on routine ultrasound examination. Our patient continued to suffer from severe bone pain and impaired mobility, with an ongoing need of the wheelchair. With the conventional supplementation therapy, we were unable to achieve serum phosphate levels above 0.6 mmol/l. After two years without satisfying therapeutic success under conventional management, and 1 month after its approval, we initiated an off-label treatment with burosumab in May 2018. Two weeks prior to the initiation of burosumab therapy, we stopped the supplementation therapy, and started with bi-weekly subcutaneous burosumab injections with a dose of 0.4 mg/kg. Clinical trials in XLH patients recommended to increase the dose to 1.2 mg/kg if the serum phosphate levels do not increase by > 0,16 mmol/l from baseline, or if 2 consecutive parameters were below the normal range (6). After 4 weeks of therapy, we observed an increase of the phosphate levels from initially 0.58 to 0.68 mmol/l which was still below the normal range of 1.05-1.8 mmol/l. We, therefore, increased the dose to 0.8 mg/kg. Serum phosphate levels then normalized and have, subsequently, remained within the age-appropriate range (**Figure 2A**). Importantly, after an initial training session by the nursing staff, parents were able to administer the injections at home, avoiding unnecessary hospital visits. Over the complete therapy course of 42 months, only 1 dose change was necessary to adjust for increasing body weight, while the dose per body weight was effectively decreased to 0.7 mg/kg in July 2020. During 42 months of treatment, the patient was closely monitored for overall clinical condition, laboratory values for mineral homeostasis, as well as radiographic imaging, and occurrence of any adverse effects. During the first six months, follow-up was performed every 4-6 weeks. As the clinical status and the laboratory parameters remained stable, we extended the follow-up intervals to 6 months. After the therapy start with burosumab serum phosphate levels increased to an age-appropriate range after only 4 weeks and remained constantly in the age-adjusted normal range between 1.0-1.8 mmol/l (**Figure 2A**). Furthermore, we achieved a satisfying serum concentration of calcitriol (62 pg/ml [22-75 pg/ml]) without an increase in ionized calcium levels [1.21 mmol/l (1.12-1.37 mmol/l)] (**Figure 2B**). PTH (**Figure 2C**) and urine calcium excretion levels remained normal. We also detected a significant decrease of alkaline phosphatase levels (from 754 μg/l to 75 μg/l [normal range 37-97 μg/l]) after only a few weeks of therapy (**Figure 2D**). The urine calcium and phosphate excretion were normal, with a normalized tubular phosphate reabsorption rate. The nephrocalcinosis and the initially observed right multicystic dysplastic kidney were unchanged on ultrasound exams. To evaluate the rickets improvement, a seven-point scale (radiographic global impression of change score (RGI-C)) was used to monitor bone structure and skeletal maturity (−3 = severe worsening, 0 = no change; +3= near/complete healing) (7). Hallmarks were the level of osteopenia, metaphyseal fraying, and irregularity of the provisional zone of calcification. According to the RGI-C score, our patient showed a substantial improvement in rickets severity (+2). A radiograph of the left hand in February 2018 demonstrated rickets with a determined bone age of 1-3 years (patient´s age 2018: 5 years). Conversely, the current radiograph, after 42 months of burosumab treatment, presented a nearly age-appropriate bone age of 7 years and 5 months (patient´s age: 8 years) (**Figures 3A, B**). In addition, the bone growth velocity increased from 5 cm per year to 7.5 cm per year. The increasing size of the thorax led to improved respiratory mechanics and termination of oxygen administration. A leading symptom before initiation of the burosumab therapy was an almost daily occurrence of bone pain that required frequent NSAID intake. This had also significantly improved with the characteristic bone pain now only occurring in the night before the next burosumab application. Originally, skeletal abnormalities and bone pain had an immense impact on the patient’s mobility. After 8 weeks of burosumab therapy, we noticed an improvement in her walking pattern, with an increase of wheelchair-free time of 1-2 hours per day. In the following year, wheelchair-free time further increased and since the patient started primary school at the age of 7 years, she only needed to use the wheelchair during some group activities in physical education class. She was able to walk for 4 to 5 consecutive hours during a weekday and 2 to 3 hours on weekends. Furthermore, she was capable to walk distances from 1 to 2 kilometers without interruption. For stairs or to cover distances beyond 2 kilometers, the patient still uses crutches. In summary, after initiation of burosumab treatment, constant improvements of the walking pattern and walking distances were observed by both the parents and physicians. To date, no adverse events occurred, especially no local reactions at the injection site (hypersensitivity, pruritus, swelling or rash) were noted. Compliance to the therapeutic regimen was high, mainly due to significant decrease in bone pain immediately after the injections.

## Discussion

Epidermal nevus syndromes are a very rare and heterogeneous group of congenital disorders that include CSHS. The CSHS phenotype is assumed to result from a multilineage involvement of a mosaic RASopathy (8). Due to the low prevalence of these syndromes, specific phenotypes are poorly characterized, and no causal therapies are established. The, here, described patient presented with a nevus sebaceous in combination with a mosaic *NRAS* variants, that is more typically detected in congenital melanocytic nevus or neurocutaneous melanosis (9). However, the same variant has also been reported in cases of CSHS with melanocytic as well as epidermal nevi (10). Furthermore, kidney abnormalities (e.g. horseshoe kidney, duplicated urinary collecting system) have occasionally been reported in patients with Schimmelpenning-Feuerstein-Mims syndrome but not in CSHS. However, the unilateral multicystic kidney could be an incidental finding and unrelated to the detected *NRAS* variant. Our patient showed severe thoracic deformity with scoliosis and developed a chronic demand for oxygen after a Legionella pneumonia. Further examinations showed the histological combination of neuroendocrine cell hyperplasia (NEH) with an alveolar hypoplasia. This combination is known to occur in patients with airway immaturity on the basis of thoracic insufficiency syndrome (11). Burosumab treatment improved the bone mineralization, increased the thoracic growth capacity and, as a consequence, reduced the thoracic insufficiency. Pulmonary symptoms are not described in CSHS patients, however it can be explained due to thoracic insufficiency syndrome and aggravated by the acute pneumonia and NEH.

Based on molecular testing results and clinical phenotype of the patient, including hypophosphatemia and bone pain, we diagnosed the observed condition as CSHS. CSHS features an elevation of the 32 kDa protein FGF23, that is predominantly expressed in bones and secreted by osteocytes. The expression of FGF23 is regulated by local (e.g. phosphate-regulating neutral endopeptidase = PHEX) and systemic (phosphate, calcitriol) bone-derived factors (12). Usually, FGF23 production is upregulated by an increase of calcitriol, serum phosphate and parathormone levels. The most important FGF23 target is the kidney, followed by the parathyroid gland. In kidneys, FGF23 suppresses the expression of certain cotransporters, mainly Na/Pi-2, that regulate phosphate uptake in renal proximal tubular epithelial cells (13). FGF23 inhibits these cotransporters which leads to a reduction of phosphate reabsorption in proximal tubules, and thereby to increased urinary phosphate loss. Additionally, FGF23 downregulates the expression of 1-alpha-hydroxylase, the enzyme synthesizing calcitriol (14). As a consequence, patients with CSHS and FGF23 excess develop severe FGF23-mediated hypophosphatemia, causing rickets, growth retardation, and bone pain (10). So far, conventional therapy of these patients solely comprised administration of oral phosphate and calcitriol. Unfortunately, previous studies suggested that conventional therapy only leads to a transient increase in serum phosphate, without affecting urine phosphate loss (15, 16). This lack of rescue is also evidenced by the persistence of skeletal abnormalities, even after prolonged treatment with phosphate and vitamin-D metabolites. The high burden of bone pain, fractures and severe rickets necessitates a causative therapeutic approach. A promising new therapeutic approach for FGF23-mediated hypophosphatemia is treatment with the monoclonal antibody burosumab (**Table 1**). Burosumab is a human, recombinant IgG antibody that binds to FGF23 and thus inhibits its excessive activity (17). In 2018, burosumab received first approval for the treatment of children > 6 months of age with X-linked hypophosphatemia (XLH) and tumor-induced osteomalacia (TIO). In randomized, controlled studies in patients with XLH, treatment with burosumab led to a normalization of serum phosphate levels, which was not observed in patients receiving conventional therapy. In the burosumab treatment group, adverse effects were observed more frequently. However, the most common adverse effects were not severe, and mainly included local reactions (hypersensitivity, pruritus, swelling or rash) at the injection site (18).

**Table 1:** Comparison of burosumab treatment regimens, dose and follow-up across pediatric and adult patients with monogenic or paraneoplastic hypophosphatemia syndromes.

Study / trial number (if applicable)	Current case report	(4) Case report Khadora et. al	(6) Imel et al. / NCT02915705	(5) De Beur et al. / UX023T‐CL201
Disease	CSHS	CSHS	X-linked hypophosphatemia (XLH)	mixed (TIO, XLH, CSHS)
**Inclusion criteria**	**conservative treatment for 24 months; severe generalized bone pain/rickets; impaired mobility (wheelchair, walking distance 15 m); phosphate levels < 0.6 mmol/l**	**conservative treatment for 12 months; incomplete healing of pseudofractures/rickets; 6 minute walk test 292m; phosphate levels 0.8 mmol/l**	**serum phosphate < 0.97 mmol/L; confirmed PHEX mutation (patient, or member of family with x linked dominant inheritance); Thacher rickets severity score of at least 2.0; conservative treatment: children < 3 years: for ≥ 6 consecutive months; children > 3 years: for 12 consecutive months**	**TIO not curable by surgical excision; serum phosphate <0.81 mmol/l; TmP/GFR <0.81 mmol/L;FGF23 ≥100 pg/mL; corrected serum calcium <10.8 mg/dL**
**Number of patients**	**1**	**1**	**29**	**17 (14 TIO, 1 CSHS, 2 XLH)**
**Age of patients**	**3 years**	**3 years, 10 months**	**1-12 years**	**≥18 years**
**Washout period after discontinuing conservative treatment**	**2 weeks**	**1 week**	**1 week**	**2 weeks**
**Initial burosumab dose**	**0.4 mg/kg**	**0.8 mg/kg**	**0.8 mg/kg**	**0.3 mg/kg**
**Dose adjustments**	**increase after 1 month to 0.8 mg/kg, after 24 months reduction to 0.7 mg/kg**	**decrease after 1 month to 0.4 mg/kg, maintained over next 12 months**	**increase up to 1.2 mg/kg if: 2 consecutive phosphate concentrations < 1.03 mmol/L, phosphate increased by only <0.16 mmol/L from baseline**	**increase until week 16, maximal dose of 2.0 mg/kg**
**Burosumab injection regimen**	**bi-weekly, subcutaneously**	**bi-weekly, subcutaneously**	**bi-weekly, subcutaneously**	**every 4 weeks, subcutaneously**
**Side effects**	**None**	**None**	**17 (59%) of 29 patients, mostly injection** **site-related (mild in severity, resolved within days); 1 patient with arthralgia (severe adverse effect), resolved within days**	**all patients ≥ 1 adverse effect (typical TIO symptoms or unspecific); 9 patients with adverse effects related to burosumab, most frequent (≥ 4 patients): pain in extremity/back, diarrhea, cough, arthralgia, neoplasm progression, muscle spasms, nasopharyngitis, upper respiratory tract infection**
**Follow-ups**	**first 6 months: every 4-6 weeks** **After 4 months every 6 months**	**not specifically described**	**Initial evaluation 2 weeks after first injection and extended follow-ups after 10 and 16 months; depending on laboratory parameters (especially serum phosphate), different individual follow-ups performed**	**Initial evaluation 2 weeks after first injection and extended follow-ups after 12 and 36 months; depending on laboratory parameters (especially serum phosphate), different individual follow-ups performed**
**Parameters monitored in all studies**	**calcium, phosphate, alkaline phosphatase, urinary phosphate, TmP/GFR, parathyroid hormone, vitamin D (25-Hydroxy and 1,25-Dihydroxy), urine calcium excretion**			
**Additional tests**	**RGI-C score, bone age, bone growth velocity, 6-min walking test**	**Pediatric Quality of Life Inventory™ 4.0 questionnaire (PedsQL4), 6-min walking test**	**RGI-C score, Thacher Rickets Severity Score, Lower limb deformity score, length/standing height Z-score (using age, sex matched normative data, US CDC), bone age, bone growth velocity, 6-min walking test**	**Bone biopsy analysis, CTX, P1NP, osteocalcin, 99mTc-MDP whole-body bone scans, Brief Pain Inventory (worst pain score, pain severity, pain interference), Global fatigue score, SF-36v2 physical component score, Sit-to-stand repetitions, 6-min walking test**
**Time course of treatment**	**42 months**	**12 months**	**4 months**	**36 months**

Each column represents a different study, using burosumab to treat hypophosphatemia and related symptoms in either a genetic syndrome (CSHS, Cutaneous-skeletal hypophosphatemia syndrome; XLH, X-linked hypophosphatemia) or paraneoplastic tumor-induced osteomalacia (TIO) (4–6). Of note, in this study of De Beur et al. one CSHS patient was included but results not reported in the present publication (5). CSHS, cutaneous-skeletal hypophosphatemia syndrome; CTX, C-terminal telopetide; PHEX Phosphate Regulating Endopeptidase Homolog X-Linked; P1NP, Procollagen type I N-terminal propeptid; RGI-C, Radiographic Global Impression of Change; SF- 36v2, specific quality of health questionnaire; TIO, tumor induced osteomalacia; XLH, X-linked hypophosphatemia; 99mTc-MDP, scinitigraphy with 99mTc-labeled methylene-diphosphonate.

Consistently with studies in XLH, the here described patient showed a significant improvement in growth, bone metabolism and rickets severity under burosumab therapy (6). Furthermore, no adverse effects were observed over the whole treatment course of 42 months while clinical improvement was consistently maintained. To our knowledge, only one similar case of a pediatric CSHS patient treated with burosumab over a time course of 12 months has been very recently reported (4). During their short-term treatment, Khadora et al. showed similar results regarding clinical outcome and an overall satisfying safety profile. In our case treatment was initiated with a dose of 0.4 mg/kg and increased to 0.8 mg/kg, while Khadora et al. started off with 0.8 mg/kg (**Table 1**). Interestingly, in both cases the weight-adjusted burosumab dose could be reduced over the course of the therapy while positive effects were maintained. In our case, the dose could be reduced to 0.7 mg/kg compared to 0.3-0.4 mg/kg in the patient described by Khadora et al. Importantly, even after long-term treatment of 42 months, no decrease in effectiveness was observed in the here described case. Further supporting data was generated in the ongoing phase 2 trial (UX023T‐CL201) in adult TIO patients, receiving burosumab treatment (**Table 1**) (5). The study also includes one adult patient with CSHS, however results for this particular patient were not included in the present publication of the study (5).

In conclusion, we, here, report the first successful long-term treatment of a pediatric CSHS patient with burosumab. Currently, patients receiving conventional therapy only show poorly improved mineral homeostasis with a persistence of clinical symptoms, severely limiting physical activity and overall development. We, here, gathered first evidence for long-term control of mineral homeostasis, bone morphology and physical activity in a pediatric patient with CSHS and treatment with burosumab. However, systematic studies are warranted to assess the efficacy and safety profile in a larger cohort of pediatric CSHS patients.

## Patient Perspective

From the initial presentation over the first 2 years with conventional treatment, the patient was never able to walk further than 10 to 20 meters and was mainly wheelchair-bound. Overall development and age-appropriate quality of life were further severely affected by very frequent rickets-associated bone pain and weak bone morphology. After initiation of the treatment with burosumab, a tremendous improvement of bone pain and walking ability was observed, while the overall participation in activities of daily living was greatly increased. Over the time course of 42 months, the patient showed constant progress in her physical development. Physical activity and pain-free mobility are cornerstones of age-appropriate development and the overall maturation process. Therefore, the improvements under burosumab treatment signify an invaluable contribution to her general development. An important additional aspect for the patient’s quality of life was the greatly improved skin condition. Under burosumab treatment, no ulceration and secretion with associated irritation or itching skin were observed.

## Data Availability Statement

The datasets presented in this article are not readily available because of ethical/privacy reasons. Requests to access the datasets should be directed to the corresponding author.

## Ethics Statement

Written informed consent was obtained from the individual's next of kin for the publication of any potentially identifiable images or data included in this article.

## Author Contributions

LM, KD, and FB contributed to conception and design of the case report. NZ and MS are current main physician, provided consent form and current data about the patient. IW and MZ performed molecular testing and confirmed diagnosis. NT and TW took care of request for burosumab therapy and the exchange with health insurance. TL and FP were in charge of pulmonary diagnostics. LM and FB wrote the first draft of the manuscript. FB wrote sections of the manuscript. LM and FB contributed to manuscript revision, read, and approved the submitted version.

## Funding

We acknowledge support from the Leipzig University for OpenAccess Publishing.

## Conflict of Interest

The authors declare that the research was conducted in the absence of any commercial or financial relationships that could be construed as a potential conflict of interest.

## Publisher’s Note

All claims expressed in this article are solely those of the authors and do not necessarily represent those of their affiliated organizations, or those of the publisher, the editors and the reviewers. Any product that may be evaluated in this article, or claim that may be made by its manufacturer, is not guaranteed or endorsed by the publisher.
